# Factors Influencing the Adjuvant Therapy Decision: Results of a Real-World Multicenter Data Analysis of 904 Melanoma Patients

**DOI:** 10.3390/cancers13102319

**Published:** 2021-05-12

**Authors:** Georg Lodde, Andrea Forschner, Jessica Hassel, Lena M. Wulfken, Friedegund Meier, Peter Mohr, Katharina Kähler, Bastian Schilling, Carmen Loquai, Carola Berking, Svea Hüning, Kerstin Schatton, Christoffer Gebhardt, Julia Eckardt, Ralf Gutzmer, Lydia Reinhardt, Valerie Glutsch, Ulrike Nikfarjam, Michael Erdmann, Andreas Stang, Bernd Kowall, Alexander Roesch, Selma Ugurel, Lisa Zimmer, Dirk Schadendorf, Elisabeth Livingstone

**Affiliations:** 1 Department of Dermatology, Venereology and Allergology, University Hospital Essen, 45147 Essen, Germany; georg.lodde@uk-essen.de (G.L.); alexander.roesch@uk-essen.de (A.R.); selma.ugurel@uk-essen.de (S.U.); lisa.zimmer@uk-essen.de (L.Z.); dirk.schadendorf@uk-essen.de (D.S.); 2 Department of Dermatology, University Hospital Tuebingen, 72076 Tuebingen, Germany; andrea.forschner@med.uni-tuebingen.de (A.F.); julia.eckardt@med.uni-tuebingen.de (J.E.); 3 Department of Dermatology, University Hospital Heidelberg, 69120 Heidelberg, Germany; Jessica.Hassel@med.uni-heidelberg.de; 4 Skin Cancer Center Hannover, Department of Dermatology and Allergy, Venereology and Allergology, University Hospital Hannover Medical School, 30625 Hannover, Germany; wulfken.lena@mh-hannover.de (L.M.W.); gutzmer.ralf@mh-hannover.de (R.G.); 5 Skin Cancer Center at the University Cancer Centre Dresden and National Center for Tumor Diseases, 01307 Dresden, Germany; Friedegund.Meier@uniklinikum-dresden.de (F.M.); lydia.reinhardt@uniklinikum-dresden.de (L.R.); 6 Department of Dermatology, Elbe Kliniken Stade-Buxtehude, 21614 Buxtehude, Germany; Peter.Mohr@elbekliniken.de; 7 Department of Dermatology, Venereology and Allergology, University Hospital Kiel, 24105 Kiel, Germany; kkaehler@dermatology.uni-kiel.de; 8 Department of Dermatology, Venereology and Allergology, University Hospital Wuerzburg, 97080 Wuerzburg, Germany; schilling_b@ukw.de (B.S.); glutsch_v@ukw.de (V.G.); 9 Department of Dermatology, University Hospital Mainz, 55131 Mainz, Germany; carmen.loquai@unimedizin-mainz.de (C.L.); ulrike.nikfarjam@unimedizin-mainz.de (U.N.); 10 Department of Dermatology, University Hospital Erlangen, CCC-Comprehensive Cancer Center Erlangen-EMN, Erlangen, Friedrich-Alexander University Erlangen-Nuremberg (FAU), Deutsches Zentrum Immuntherapie (DZI), 91054 Erlangen, Germany; Carola.Berking@uk-erlangen.de (C.B.); michael.erdmann@uk-erlangen.de (M.E.); 11 Department of Dermatology, Klinikum Dortmund gGmbH, 44137 Dortmund, Germany; svea.huening@klinikumdo.de; 12 Department of Dermatology, Medical Faculty, Heinrich Heine University, 40225 Düsseldorf, Germany; kerstin.schatton@med.uni-duesseldorf.de; 13 Department of Dermatology, Venereology and Allergology, University Hospital Hamburg, 20246 Hamburg, Germany; ch.gebhardt@uke.de; 14 Institute for Medical Informatics, Biometry and Epidemiology, University Hospital Essen, 45122 Essen, Germany; imibe.dir@uk-essen.de (A.S.); Bernd.Kowall@uk-essen.de (B.K.); 15 German Consortium for Translational Cancer Research (DKTK), Partner Site Essen and German Cancer Research Center (DKFZ), 45147 Essen, Germany

**Keywords:** melanoma, adjuvant treatment, checkpoint blocker, targeted therapy, *BRAF*, PD-1

## Abstract

**Simple Summary:**

Adjuvant treatment of stage III/IV melanoma patients with immune-checkpoint inhibition or targeted therapy can significantly improve recurrence-free survival. However, it is unknown how many patients with an indication for adjuvant therapy do indeed choose to receive it and what the reasons for declining are. In patients with a *BRAF* mutation, it is not known whether more patients prefer targeted or immunotherapy. This study investigates the real-world situation of 904 patients from 13 German Dermatologic Cooperative Oncology Group skin cancer centers with an indication for adjuvant treatment since the approval of the corresponding drugs as adjuvant treatment. Aims of this study were to investigate the patient groups who opt for or against adjuvant treatment, respectively targeted or immunotherapy, and the reasons for refusal. Findings of this study show the current acceptance and choice of adjuvant melanoma treatment and may support patients and physicians in the therapy decision-making process.

**Abstract:**

Adjuvant treatment of melanoma patients with immune-checkpoint inhibition (ICI) and targeted therapy (TT) significantly improved recurrence-free survival. This study investigates the real-world situation of 904 patients from 13 German skin cancer centers with an indication for adjuvant treatment since the approval of adjuvant ICI and TT. From adjusted log-binomial regression models, we estimated relative risks for associations between various influence factors and treatment decisions (adjuvant therapy yes/no, TT vs. ICI in *BRAF* mutant patients). Of these patients, 76.9% (95% CI 74–80) opted for a systemic adjuvant treatment. The probability of starting an adjuvant treatment was 26% lower in patients >65 years (RR 0.74, 95% CI 68–80). The most common reasons against adjuvant treatment given by patients were age (29.4%, 95% CI 24–38), and fear of adverse events (21.1%, 95% CI 16–28) and impaired quality of life (11.9%, 95% CI 7–16). Of all *BRAF*-mutated patients who opted for adjuvant treatment, 52.9% (95% CI 47–59) decided for ICI. Treatment decision for TT or ICI was barely associated with age, gender and tumor stage, but with comorbidities and affiliated center. Shortly after their approval, adjuvant treatments have been well accepted by physicians and patients. Age plays a decisive role in the decision for adjuvant treatment, while pre-existing autoimmune disease and regional differences influence the choice between TT or ICI.

## 1. Introduction

Melanoma is one of the most aggressive skin cancers worldwide. Its incidence has increased over the last 30 years, and a further increase is predicted [1]. More than 230,000 melanomas are diagnosed annually [1,2]. Rates are highest in Australia (age-standardized rate per 100,000: 33.6), but while Australia is one of the few countries where incidence rates seem to be declining [2], numbers are still rising in most other countries, including Germany (age-standardized rate per 100,000: 21.6) [3,4].

Melanoma is classified into prognostic subgroups according to characteristics of the primary tumor, as well as the presence of locoregional and of distant metastases [5]. Patients in stages III (locoregional metastasis) and IV (distant metastasis) have a high risk of relapse and melanoma-specific death after complete tumor resection [5,6,7,8]. Adjuvant treatment with the aim of improving prognosis is therefore a relevant strategy in melanoma therapy.

Randomized controlled phase 3 trials demonstrated significantly improved recurrence-free survival in melanoma patients treated with adjuvant immune-checkpoint inhibition (ICI) or targeted therapy (TT) after complete tumor excision [9,10,11,12]. Due to the positive trial results, the anti-PD-1 antibodies nivolumab and pembrolizumab and the BRAF inhibitor dabrafenib, in combination with the MEK inhibitor trametinib, were approved for adjuvant treatment of melanoma patients by both the U.S. Food and Drug Administration (FDA) and the European Medicines Agency (EMA). Nivolumab was approved for patients in stages III and IV after complete resection by EMA on 30 July 2018. Pembrolizumab is restricted to patients in stage III after complete resection of lymph node metastases and was approved by EMA on 17 December 2018. On 29 August 2018, EMA approved dabrafenib and trametinib for stage III *BRAF V600* mutated melanoma patients after complete resection of metastases. Before the era of adjuvant ICI and TT, adjuvant systemic therapy for melanoma consisted of interferon α with high toxicity and limited effect on overall survival [13,14].

ICI is administered as intravenous infusions either every two or every four weeks (nivolumab) or every three or six weeks (pembrolizumab) for one year in the adjuvant setting. In melanoma, ICI can be used irrespective of PD-L1 or mutational status. However, ICI can induce immune-related adverse events [15,16] ranging from mild rash to severe; e.g., colitis, hepatitis or pneumonitis, with indication for inpatient care and a potentially fatal outcome. In the adjuvant trials CheckMate238 and KEYNOTE-054, grade 3–4 events occurred in 14.4–14.7% of nivolumab- or pembrolizumab-treated patients [16,17]. Immune-related adverse events are treated with systemic corticosteroids and, if required, other immunosuppressants over a longer period. Endocrine adverse events can lead to a permanent hormone deficiency, requiring lifelong substitution. Most adverse events develop in the first weeks of treatment, but late-onset events occurring months after the last dose are possible, albeit rare [9,10]. Treatment discontinuation due to treatment-related adverse events was reported in 7.7–13.0% in the adjuvant setting [16,17] One treatment-related death as a result of myositis was reported in a pembrolizumab-treated patient [17].

Dabrafenib plus trametinib is a treatment option for patients with a *BRAFV600* mutation, which is present in about 50% of cutaneous melanomas [2]. In the adjuvant setting, patients receive oral dabrafenib 150 mg BID and trametinib 2 mg QD for one year, which is equivalent to five tablets per day. Patients can take the tablets on their own, however, an interval of one hour before or two hours after a meal is required, and trametinib tablets need to be refrigerated at 2–8 °C. TT displays a range of adverse events; the most common are pyrexia, nausea, fatigue, headache and chills [18]. Discontinuation due to an adverse event occurred in 26% of patients in the adjuvant COMBI-AD trial [18]; however, adverse events of TT generally seize rapidly after stopping TT without additional treatment. No treatment-related deaths were reported in COMBI-AD trial.

In Germany, adjuvant treatment with ICI and TT outside of clinical trials has been possible since the day of EMA authorization. Unlike clinical trials, which have distinct inclusion and exclusion criteria and rigid time schedules, adjuvant treatment in daily clinical practice is primarily dependent on indication and contraindication. Patients and physicians must weigh the advantages and risks of adjuvant therapy, but ultimately the decision is made by the patient after having been informed about the treatment options. The percentage of patients with an indication for systemic adjuvant treatment who opt for or against it, as well as the reasons for declining adjuvant treatment, are unknown. Melanoma patients with a *BRAFV600* mutation additionally have the choice between ICI and TT. Head-to-head clinical trial data of adjuvant ICI and TT do not exist, and no clear benefit of one treatment in terms of relapse-free or overall survival can yet be deducted from current clinical trials [9,10,11]. Decisions for or against the specific adjuvant treatment are therefore dependent on patients’ preferences such as mode of application, frequency of patient visits or potential toxicity.

The aim of this multicenter, retrospective cohort study was to characterize the populations of stage III and IV melanoma patients with an indication for adjuvant treatment who opt for or against adjuvant systemic treatment, and in *BRAFV600* mutated patients who decide for ICI or TT. We investigated the relationship between patient characteristics and (1) the decision for or against adjuvant systemic treatment, and in patients with a *BRAFV600* mutation (2) the choice of adjuvant treatment type. Additionally, patients’ reasons against adjuvant therapy were documented.

## 2. Materials and Methods

### 2.1. Patient Selection

All melanoma patients in stages III and IV ≥18 years without evidence of disease after complete tumor resection and an indication for adjuvant systemic treatment between 1 June 2018 and 30 September 2019 were eligible irrespective of whether they started adjuvant treatment or not. The start of recruitment was chosen two months before the approval of ICI or TT as adjuvant treatment, as the EMA had already officially recommended approval. At this point, tumor boards had begun recommending adjuvant ICI or TT treatment, with start of treatment after full approval. The participating centers (*n* = 13) were skin cancer centers of the German Dermatologic Cooperative Oncology Group (DeCOG) with high treatment and documentation standards (Figure S1). Data on gender, age at diagnosis of stage III/IV, comorbidities, date of diagnosis, location of primary, histological subtype, mutation status (*BRAF, NRAS, KIT*), date of first stage III/IV, previous therapies including completion lymph node dissection and radiation, reasons against adjuvant treatment given by patients and start of adjuvant treatment were collected by each cancer center. The American Joint Committee on Cancer (AJCC) classification 8th edition was used as the cancer-staging system. Patients who had received prior systemic adjuvant treatment and experienced a relapse while on or after prior adjuvant treatment received a complete resection and subsequently were offered another adjuvant treatment with TT or ICI. To quantify comorbidity, we used the modified Charlson comorbidity index (CCI) developed by Quan et al. in 2005 [19]. It includes 17 comorbidities weighted originally by Charlson et al. in 1987 [20]. Theses comorbidities are displayed according to the ICD-10 code (Table S1). The modified CCI was used with the exclusion of melanoma as a relevant malignant comorbidity, as every patient by definition had a malignancy (melanoma).

Data were extracted from patient records at the respective institution and merged centrally for analysis. Patients were excluded if they received adjuvant treatment in clinical trials. The study was approved by the institutional ethics committee of the University Duisburg-Essen (BO-19-8863) and by the respective local ethics committees as necessary.

### 2.2. Statistical Analysis

To compare groups, clinical and demographic patient characteristics were evaluated. Numerical variables were described by median and range. Patient cohorts were compared using a t-test and two-sided χ2 or Fisher’s exact tests, as appropriate.

For causal modeling, we performed log-binomial regression analyses to test possible associations between the collected variables and the decision for or against adjuvant treatment. In patients whose *BRAFV600* mutation status was known at the time of treatment decision, associations were additionally studied for the decision for TT or ICI. The following variables were included: age at time of decision (≤65 years (reference, ref.) vs. >65 years), gender (female (ref.) vs. male), *BRAF*-mutation status (wild type (ref.) vs. *BRAF*-mutation), Charlson comorbidity index (CCI 0 (ref.) vs. CCI ≥ 1), autoimmune disease (no (ref.) vs. yes), region of skin cancer center (Northern Germany: Kiel, Hamburg, Buxtehude, Hannover, Dortmund, Essen, Düsseldorf, Dresden (ref.) vs. Southern Germany: Mainz, Heidelberg, Erlangen, Würzburg, Tübingen), health insurance status (statutory health insurance (ref.) vs. private health insurance), prior adjuvant systemic therapy (no (ref.) vs. yes) and tumor stage at time of decision (IIIA, IIIB, IIIC, IIID, IV). To define the confounder adjustment sets, directed acyclic graphs (DAG) [21] were created (Figures S2 and S3). DAGs were created according to consensus from melanoma experts from different skin cancer centers, as well as empiric literature research. Confidence intervals were calculated and reported to assess the precision of estimates [22]. When variables of the confounder adjustment set were missing, regression analyses with multiple imputation were performed in addition to regression analyses with complete cases. Missing data were imputed with PROC MI in SAS 9.4, edition 9.4, SAS Institute, Cary, North Carolina, USA.

Statistical analyses were performed using IBM SPSS Statistics software (version 26.0, International Business Machines, Armonk, NY, USA) and SAS (edition 9.4, SAS Institute, Cary, NC, USA).

## 3. Results

Data from 941 patients from 13 national skin cancer centers with an indication for adjuvant treatment of resected stage III or IV melanoma were entered into the central registry. Three patients were excluded due to age (<18 years); 34 patients from a single center were only included in the analysis of choice between TT and ICI, as data on patients who did not opt for adjuvant treatment were not available from this center, leaving 904 patients for the main analysis (see flowchart of patient inclusion; Figure S4). Median age at the time of decision for or against adjuvant treatment was 64.9 years (range 19.2–98.1; Table 1). There was a small male predominance (58.2%; *n* = 526); 26.1% of the patients (*n* = 236) had a modified CCI score of 1 or 2, and 5.2% (*n* = 47) had 3 or more relevant comorbidities. A *BRAF* mutation was detected in 35.4% of patients (*n* = 320). In 9.2% of patients (*n* = 83), a mutational analysis was only performed after start of adjuvant therapy due to, e.g., insufficient amount of tumor material for mutational analysis in the sentinel node. In these patients, mutational analysis was only performed when the centers received tumor material from external pathologies (mostly primary tumor) or at the time of relapse. Almost half of the patients (47.1%, *n* = 426) were classified as stage IIIC, and 32.3% (*n* = 292) had stage IIIB AJCC8 disease. Stage IV NED comprised only 79 patients (8.7%).

In 38 cases, the tumor board decided against recommending adjuvant treatment due to the patient´s comorbidities (*n* = 33) or as the tumor burden in the sentinel node was <0.1 mm (*n* = 5). All patients for whom the tumor board decided against adjuvant therapy due to comorbidities were >65 years. One patient experienced progression between the tumor board’s decision and patient discussion of adjuvant therapy recommendation.

### 3.1. Decision on Adjuvant Treatment

In total, 695 patients (76.9%; 95% CI 74–80) opted for a systemic adjuvant treatment. Patients who decided against adjuvant treatment were markedly older (median age 76.3 years (range 27.7–98.1) vs. 61.8 years (19.2–89.9)) and had more comorbidities (CCI 0 57.4% (95% CI 51–64) vs. 72.0% (95% CI 69–75)). Barely any gender differences were noted. More patients in tumor stage IIID (88.5%, 95% CI 76–100) and fewer patients in stage IIIA opted for adjuvant treatment (71.6%, 95% CI 62–81). In all other tumor stage subgroups, rates were comparable. *BRAF*-mutant patients (83.4%, 95% CI 79–87), patients with a prior completion lymph node dissection (81.7%, 95% CI 77–86), prior adjuvant systemic, local or combined systemic plus local therapy (87.7%, 95% CI 81–93), or radiotherapy (82.2%, CI 76–88) were more likely to opt for adjuvant treatment compared with the overall cohort. The most commonly used prior systemic adjuvant therapy was interferon (*n* = 88). Only 11 patients had received prior adjuvant treatment with TT (*n* = 5), a CTLA-4 (*n* = 2), anti-PD-1 (*n* = 2) or anti-PD-1 inhibitor in combination with an intratumoral injection (*n* = 2). The rate of patients who opted for or against adjuvant systemic treatment per center is depicted in Figure S5.

The most common reasons cited by patients against adjuvant treatment were age (29.4%, 95% CI 24–38), fear of adverse events (21.1%, 95% CI 16–28), fear of impaired quality of life (11.9%, 95% CI 7–16) and too high an effort (6.9%, 95% CI 4–11) (Table 2). Most patients naming these four reasons were >65 years (*n* = 118); only 33 patients (15.1%) who had given at least one of these reasons were ≤65 years (Table S2). Female patients more frequently named fear of adverse events (29.5%, 95% CI 20–40) than male patients (14.6%, 95% 9–23).

DAG-adjusted log-binomial regression models showed that age was the only variable that was significantly associated with the decision for or against a systemic adjuvant treatment. The probability of starting an adjuvant treatment was 26% lower in patients >65 years compared to patients ≤65 years (age >65 vs. age ≤65 (ref.), relative risk (RR) 0.74, 95% CI 0.68–0.80; Table 3). The adjusted model barely showed any difference between patients with or without comorbidities (modified CCI ≥1 vs. 0 (ref.), RR 0.99, 95% CI 0.93–1.05), with a *BRAF* mutation compared to *BRAF* wild type (*BRAF* mutation vs. *BRAF* wild type (ref.), RR 1.02, 95% CI 0.97–1.07), with prior local/systemic treatment (yes vs. no (ref.), RR 1.03, 95% CI 1.03 (0.97–1.09), in stage IIIB–D versus IIIA (IIIB vs IIIA (ref.), RR 1.04, 95% CI 0.96–1.13; IIIC vs. IIIA (ref.), RR 1.04, 95% CI 0.96–1.13; IIID vs. IIIA (ref.) 1.07, 95% CI 0.89–1-30; IV vs. IIIA (ref.), RR 1.04 (0.93–1.16)).

### 3.2. Treatment Choice between Targeted and Immunotherapy in BRAF-Mutant Patients

The cohort was enriched by 13 patients from a skin cancer center that could not provide data on patients who had opted against adjuvant treatment (Figure S4). Due to the exclusive approval of adjuvant TT for stage III, patients with tumor stage IV were excluded. Characteristics of the cohort of 263 *BRAF*-mutant patients are depicted in Table 4. The rate of decision for adjuvant treatment was higher in the group of *BRAF*-mutant patients (83.4%, 95% CI 79–87, *n* = 267) than in the total cohort (76.9%, 95% CI 74–80, *n* = 695; Table 1). *BRAF*-mutant patients receiving adjuvant therapy were younger than the total cohort of adjuvant treated patients (56.3 (range 21.0–87.4) vs. 61.8 (range 19.2–89.9)). Of all *BRAF*-mutant patients who decided for adjuvant treatment, 52.9% (95% CI 47–59, *n* = 139) chose ICI, and 47.1% (95% CI 41–53, *n* = 124) chose TT. Patients with prior systemic, local or combined local plus systemic therapies (64.1%, 95% CI 47–79), with increasing tumor stage (IIIA 41.7% (95% CI 22–63) vs. IIID 54.5% (95% CI 25–86)) and with autoimmune diseases (76.5%, 95% CI 53–95) opted more often for TT than for ICI. Patients with a prior completion lymph node dissection (56.8%, 95% CI 48–66), adjuvant radiotherapy (64.3%, 95% CI 50–79) and from Southern German centers (66.1%, 95% CI 57–75) opted more often for ICI than for TT. The choice of ICI or TT per center is shown in Figure S6. No relevant differences with regard to age, gender or modified CCI was seen.

Preexisting autoimmune diseases (autoimmune disease yes vs. no (ref.), RR 1.73, 95% CI 1.25–2.39) increased the probability for opting for TT (Table 5), but the number of patients with pre-existing autoimmunity was low.

Patients treated in a Southern German skin cancer center (South vs. North (ref.), RR 0.58, 95% CI 0.44–0.77) opted less often for TT compared to patients treated in Northern German skin cancer centers (Table 5). Patients with a history of prior adjuvant treatment had a slightly higher probability to choose TT over ICI (prior adjuvant treatment yes vs. no (ref.), RR 1.24, 95% CI 0.93–1.68).

## 4. Discussion

This multicenter, retrospective cohort study with more than 900 patients shows a high acceptance of adjuvant systemic treatment in the real-world setting for stage III/IV melanoma patients. This study reveals relevant reasons given by patients for declining adjuvant treatment, as well as the choice of *BRAF*-mutated patients for ICI or TT. Until now, real-world data of adjuvant treatment of melanoma have been missing. To our knowledge, this is the first study of its kind.

Randomized trials showed a significant benefit in relapse-free survival for melanoma patients treated with adjuvant ICI or TT [9,10,11]. However, it is not known how many patients with a general indication for adjuvant therapy indeed start adjuvant therapy in the real world. Patients with a *BRAF* mutation additionally have the choice between ICI and TT. Study results to date have not demonstrated a clear advantage of one therapy over the other in the adjuvant setting, and decisions should be made according to patient preference.

Our study cohort comprised 904 melanoma patients from 13 German skin cancer centers who had an indication for adjuvant treatment since the approval of ICI and TT for the adjuvant setting. Compared with data from the preapproval era, the proportion of patients deciding for systemic adjuvant treatment was noticeably higher. While in our cohort, 76.9% of opted for adjuvant treatment, only 25.8% of German melanoma patients with stage III previously received systemic adjuvant therapy [23]. In a French retrospective analysis, only 13% of high-risk stage IIID patients received interferon-α prior to the approval of ICI and TT [24]. The improved RFS [9,10,18], as well as the experience in adverse-event management already gained in the advanced metastatic setting, might have been reasons for the rapid and high acceptance of adjuvant ICI and TT by physicians. A recent Canadian cross-sectional survey confirmed that patients considered RFS the most important and OS the second most important attribute of adjuvant treatment, and preferred active treatment to follow-up alone [25]. In our cohort, the tumor board decided against adjuvant treatment only in very few cases due to patients´ comorbidities or low sentinel tumor burden, reflecting physicians’ attitudes toward the safety and efficacy of current adjuvant therapy options.

Despite several differences between the cohorts opting for or against adjuvant treatment, age was the only variable that showed a significant association with the decision for adjuvant treatment in the DAG-adjusted regression models. In our study, patients older than 65 years had a 26% relative lower probability to start an adjuvant treatment. Other variables such as comorbidities, *BRAF* status and prior additional treatment (either as completion lymph node dissection, adjuvant systemic or local therapy) did not demonstrate a significant impact. Age, however, is associated with these variables—older patients tend to have more comorbidities [26,27,28], are less likely to be *BRAF* mutated [29,30,31] and are less likely to agree to further therapy due to their shorter life expectancy. Older age was also the most common reason given by patients for declining adjuvant therapy. Interestingly, fear of adverse events as well as quality of life was proportionally named less often by patients >65 years than by younger patients. In contrast, another German study indicated that older melanoma patients in particular feared adverse events when considering treatment in the adjuvant or metastatic setting [32]. Fear of adverse events was the most common reason given by female patients (29.5%), named almost twice as often than by male patients (14.6%), although current toxicity data in ICI and TT reveals no gender differences [33,34]. A pooled analysis of SWOG trials, however, showed that female patients experienced more subjective severe (grade 3) adverse events for ICI and TT, but objective adverse events were only more frequent for ICI [35]. Gender differences should be taken into account when counseling patients about adjuvant treatment options, with special emphasis on informing potential toxicity and the possibilities of adverse-event management of the drugs [36]. Male patients are more likely to be diagnosed with a higher tumor stage, which we could also see in our cohort (stage IIIC, male 50.6% vs. female 41.6%; IIID male 4.0% vs female 1.5%), and thus poorer prognosis [37,38]. Yet, there was no significant difference in choice of adjuvant treatment between genders.

Stage IIIA patients opted only slightly more often against systemic adjuvant therapy (71.6%) than stage IIIB (77.4%) and IIIC patients (77.2%). We had expected a significantly lower rate, as stage IIIA patients have a low recurrence risk—especially when the tumor burden of the sentinel lymph node (SLN) is <0.1 mm [39,40] and has a very good survival prognosis [5,7,8]. Both TT and ICI demonstrated a RFS benefit for stage IIIA patients in COMBI-AD and KEYNOTE-054. However, in both trials the AJCC 7th edition was used, a minimum metastatic tumor burden of the SLN of 1 mm diameter was required and SLN-positive patients had a completion lymphadenectomy, which is no longer the standard of care. KEYNOTE-054 and COMBI-AD have been reanalyzed using the AJCC 8th edition. No significant RFS benefit for AJCC 8th stage IIIA patients was seen [41,42], but the informative value of the data was limited, as a maximum of only 50 patients remained in stage IIIA. The current ESMO consensus conference stated that there is currently insufficient evidence to support the routine use of adjuvant therapy in AJCC8 stage IIIA melanoma, but that there may be some subsets of stage IIIA patients with a higher risk of relapse (e.g., tumor burden in sentinel node >1 mm) [43]. The ESMO consensus conference therefore recommended a balanced discussion of risk reduction and long-term side-effects of adjuvant therapy in these patients. In line with this, the current NCCN guideline recommends adjuvant treatment only for stage IIIA patients with at least one nodal metastasis >1 mm or stage IIIB/C, as defined by AJCC7 (TT, pembrolizumab) or stage IIIB/C (Nivolumab) [44].

Stage III patients with a *BRAF* mutation chose ICI over TT in 52.9% of cases. The rate of patients opting for TT increased with tumor stage. This trend was also found in the DAG-adjusted regression models. Clinical trial data to date have not shown that TT or ICI provide a stage-dependent benefit over the other respective drug [9,45,46,47]. TT can better prevent early relapse, as can be seen when comparing 12-month RFS rates of *BRAF*-mutant patients from COMBI-AD (88%) and KEYNOTE-054 (75.4%) [17,18]. As a rapid relapse occurs, especially in higher disease stages, patients and physicians might have opted for TT more often in higher than in lower stages. However, we had anticipated that TT, as an oral drug that offers flexibility and whose side effects generally resolve after treatment discontinuation, would be the preferred option in lower tumor stages where toxicity outweighs the risk of recurrence. The results of the DAG model demonstrated that factors other than stage played a more important role in the decision for TT or ICI.

Patients with prior adjuvant treatment, specifically prior interferon treatment, preferred TT over ICI. Systemic treatment with interferon is associated with high toxicity also of the immune-mediated spectrum and low impact on overall survival [13,14,48,49]. Patients previously treated with interferon may therefore prefer TT due to the different spectrum of adverse event and their overall reversibility after treatment discontinuation [11,18]. Three patients each had received prior ICI or TT. Of the prior TT-treated patients, two received ICI, and one was rechallenged with TT. All patients with prior ICI started TT treatment. Completion lymphadenectomy and locoregional radiotherapy had been received more often in patients opting for ICI than for TT.

In the DAG-adjusted regression models, region and autoimmune disease were the only variables that significantly impacted the choice of treatment, but the number of patients with pre-existing autoimmune disease was small. Patients treated at Southern German skin cancer centers chose TT less often compared to patients from Northern German skin cancer centers (33.9% vs. 58.5%). Regional differences, such as the distance of the patient to the treating center, but also the treating physician, had a great influence on the patient’s choice of therapy [50,51,52,53]. Rural geographical locations may be a barrier for ICI, where close follow-up of adverse events and regular visits to the site are required. In breast cancer, a lower rate of systemic treatment was seen in rural areas [54,55]. Flare of a pre-existing autoimmune disorder is common with ICI treatment [56,57,58], but information on patients with autoimmune disorder receiving ICI is only available from retrospective studies, as patients were excluded from clinical trials [56]. Additionally, in most studies heterogenous autoimmune disorders were summarized. The type, the activity and the required immunosuppression of the pre-existing autoimmune disorder need to be considered when patients are counseled on adjuvant treatment choices. A close interdisciplinary collaboration for managing ICI toxicity is recommended [59]. Patients need to be informed that even quiescent rheumatoid arthritis can flare during ICI treatment [60], and that symptoms may persist after treatment discontinuation. Increasing experience of ICI use in patients with autoimmune disorders will help to better delineate which patients can safely receive ICI.

The results of this study were limited due to the study´s retrospective nature. All information was retrieved from the hospitals´ data sources, and a centralized review was performed to minimize possible incorrect medical records and to reduce data heterogeneity. As the participating centers were all large skin cancer centers, generalizability of results to all melanoma patients in the adjuvant situation may therefore be limited. A possible influence of the physician informing the patient about his/her treatment options could not be assessed. However, this was beyond the scope of this study, the aim of which was to present the current treatment situation of melanoma patients with an indication of adjuvant therapy treated at large German skin cancer centers. Information on why patients opted for ICI or TT could not be collected, as this was generally not documented. With the inclusion of 904 patients from 13 centers, we were able to assess a large number of patients treated at different institutions by several physicians, reflecting the real-world adjuvant treatment situation since the approval of ICI and TT.

## 5. Conclusions

This study confirmed a high acceptance of ICI and TT as adjuvant treatment by both physicians and patients. Older age was the main reason for patients to decline adjuvant treatment, while other factors such as tumor stage, gender and comorbidities played a minor role in the decision-making process. Female patients especially named fear of adverse events as a reason against adjuvant treatment. Patients with a *BRAF* mutation opted for ICI slightly more often than for TT. Treatment selection was highly biased by physicians’ preference. All patients are currently being followed for adverse events, melanoma recurrence patterns, vital status and subsequent treatment, which will be reported at a later time point.

## Figures and Tables

**Table 1:** Patient characteristics of the total cohort at the time of decision for or against systemic adjuvant treatment.

Characteristics	Total Cohort		Adjuvant Treatment Cohort		No Adjuvant Treatment Cohort		*p*-Value ^e^
	*n*	%	*n*	% (95% CI)	*n*	% (95% CI)	-
-	904	100.0	695	76.9 (74.3–79.6)	209	23.1 (20.4–25.7)	-
Median age (range)	64.9 (19.2–98.1)	-	61.8 (19.2–89.9)	(60–62)	76.3 (27.7–98.1)	(70–74)	<0.001
Gender	-	-	-	-	-	-	0.92
Female	378	41.8	290	76.7 (72.2–81.1)	88	23.3 (18.9–27.8)	-
Male	526	58.2	405	77.0 (73.2–80.5)	121	23.0 (19.5–26.8)	-
Charlson comorbidity index	-	-	-	-	-	-	0.01
0	621	68.7	501	80.7 (77.5–83.8)	120	19.3 (16.2–22.5)	-
1–2	236	26.1	164	69.5 (64.1–75.6)	72	30.5 (24.4–35.9)	-
3–4	41	4.5	26	63.4 (47.8–78-0)	15	36.6 (22.0–522)	-
>5	6	0.7	4	66.7 (25.0–100.0)	2	33.3 (0.0–75.0)	-
Autoimmune disease	-	-	-	-	-	-	0.70
Yes	57	6.3	45	78.9 (68.2–88.9)	12	26.7 (11.1–31.8)	
No	847	93.7	650	76.7 (73.7–79.4)	197	23.3 (20.6–26.3)	
Tumor stage	-	-	-	-	-	-	0.48
IIIA	81	9.0	58	71.6 (61.7–81.0)	23	28.4 (19.0–38.3)	-
IIIB	292	32.3	226	77.4 (72.4–82.2)	66	22.6 (17.8–27-6)	-
IIIC	426	47.1	329	77.2 (73.3–81.0)	97	22.8 (19.0–26.7)	-
IIID	26	2.9	23	88.5 (75.9–100.0)	3	11.5 (0.0–24.0)	-
IV	79	8.7	59	74.7 (64.4–83-8)	20	25.3 (16.2–35.6)	-
Location of primary	-	-	-	-	-	-	0.93
Head/Neck	136	15.0	99	72.8 (63.3–80.0)	37	27.2 (20.0–36.2)	-
Upper extremity	137	15.2	107	78.1 (70.8–84.3)	30	21.9 (15.7–29.2)	-
Lower extremity	218	24.1	164	75.2 (69.6–81.4)	54	24.8 (18.6–30.4)	-
Trunk	297	32.9	232	78.1 (73.2–82.5)	65	21.9 (17.5–26.8)	-
Genital	13	1.4	10	76.9 (50.0–100.0)	3	23.1 (0.0–50.0)	-
Acral	38	4.2	32	84.2 (71.8–94.9)	6	15.8 (5.1–28.2)	-
Uveal	3	0.3	2	66.6 (n.e.)	1	33.3 (n.e.)	-
Other	9	1.0	7	77.8 (45.6–100.0)	2	22.2 (0.0–54.4)	-
Unknown	53	5.9	42	79.2 (69.2–90.0)	11	20.8 (10.0–30.8)	-
Histological subtype	-	-	-	-	-	-	0.42
Superficial spreading melanoma	229	25.3	179	78.2 (72.7–83.5)	50	21.8 (16.5–27.3)	
Nodular melanoma	278	30.8	208	74.8 (70.0–79.7)	70	25.2 (20.3–30.0)	-
Acrolentiginous melanoma	46	5.1	38	82.6 (70.9–92.9)	8	17.4 (7.3–28.3)	-
Lentigo maligna melanoma	27	3.0	17	63.0 (44.4–79.3)	10	37.0 (20.7–55.6)	-
Desmoplastic melanoma	13	1.4	7	53.8 (25.0–83.3)	6	46.2 (16.7–75.0)	-
Amelanotic melanoma	25	2.8	21	84.0 (66.7–96.4)	4	16.0 (3.6–33.3)	-
Mucosal melanoma	18	2.0	14	77.8 (55.6–94.7)	4	22.2 (5.3–44.4)	-
Uveal melanoma	3	0.3	2	66.7 (n.e.)	1	33.3 (n.e.)	-
Melanoma of unknown primary	72	8.0	56	77.8 (68.0–87.3)	16	22.2 (12.7–32.0)	-
Other/unknown	193	21.3	153	79.3 (73.6–85.2)	40	20.7 (14.8–26.4)	-
*BRAF* mutation status	-	-	-	-	-	-	-
Mutated	320	35.4	267	83.4 (79.2–87.3)	53	16.6 (12.7–20.8)	<0.001
Wild type	471	52.1	354	75.2 (71.0–78.8)	117	24.8 (21.2–29.0)	-
Unknown ^a^	30	3.3	22	73.3 (57.1–88.2)	8	26.7 (11.8–42.9)	-
Not specified yet ^b^	83	9.2	52	62.7 (52.2–72.3)	31	37.3 (27.7–47.8)	-
Prior intratumoral/adjuvant systemic treatment	-	-	-	-	-	-	0.002
No	782	86.5	588	75.2 (72.1–78.1)	194	24.8 (21.9–27.9)	-
Yes	122	13.5	107	87.7 (81.0–93.4)	15	12.3 (6.6–19.0)	-
Interferon	98	10.8	88	89.8	10	10.2	-
Dabrafenib/trametinib	5	0.6	4	80.0	1	20.0	-
Ipilimumab	2	0.2	2	100.0	0	0.0	-
Anti-PD1	5	0.6	5	100.0		0.0	-
Intratumoral injection ^c^	12	1.3	8	66.7	4	33.3	-
Lymphadenectomy	-	-	-	-	-	-	0.05
Yes	356	39.4	291	81.7 (77.3–85.6)	65	18.3 (14.4–22.7)	-
No	548	60.6	404	73.7 (79.8–77.3)	144	26.3 (22.7–30.2)	-
Adjuvant radiotherapy	-	-	-	-	-	-	0.09
Yes	157	17.4	129	82.2 (76.1–88.1)	28	17.8 (11.9–23.9)	-
No	747	82.6	566	75.8 (72.6–78.8)	181	24.2 (21.2–27.4)	-
Region ^d^	-	-	-	-	-	-	0.49
Northern Germany	444	49.1	337	75.9 (71.9–79.9)	107	24.1 (20.1–28.1)	-
Southern Germany	460	50.9	358	77.8 (74.0–81.5)	102	22.2 (18.5–26.0)	-
Insurance status	-	-	-	-	-	-	0.15
Statutory health insurance	594	65.7	446	75.1 (71.5–77.5)	148	24.9 (21.5–28.5)	-
Private health insurance	127	14.0	103	81.1 (74.3–87.7)	24	18.9 (12.3–25.7)	-
Insurance status missing	183	20.2	146	79.8 (74.0–85.7)	37	20.2 (14.3–26.0)	-

Abbreviations: n.e., not estimable. ^a^ Decision for adjuvant therapy (yes/no) was made without prior molecular pathology, and *BRAF* status was not routinely determined. ^b^ Mutational analysis was only performed after start of adjuvant therapy; these patients were not included in analysis of *BRAF*-mutant subgroup. ^c^ Intratumoral injection with interleukin-2, talimogene laherparepvec. ^d^ Northern German skin cancer centers: Kiel, Hamburg, Buxtehude, Hannover, Dortmund, Essen, Düsseldorf, Dresden; Southern German skin cancer centers: Mainz, Heidelberg, Erlangen, Würzburg, Tübingen. ^e^ *t*-test for continuous variables, two-sided χ2 for categorical variables or Fisher’s exact tests, as appropriate.

**Table 2:** Patients´ reasons to decline systemic adjuvant treatment.

Reasons	Cohort Refusing Adjuvant Treatment		Subgroup of *BRAF*-Mutated Patients	
	*n*	% (95% CI)	*n*	% (95% CI)
Total	218	-	59	-
Age	64	29.4 (24.4–37.5)	12	20.3 (7.7–28.6)
Fear of adverse events	46	21.1 (16.3–27.9)	15	25.4 (16.7–41.0)
Fear of impaired quality of life	26	11.9 (7.4–16.0)	7	11.9 (3.5–21.2)
Too much effort	15	6.9 (3.5–10.0)	7	11.9 (3.5–21.2)
Low risk of recurrence (patient opinion)	11	5.0 (2.5–8.8)	3	5.1 (0.0–10.2)
Time interval between initial diagnosis of melanoma and recurrence	6	2.8 (1.0–5.9)	2	3.4 (0.0–9.3)
Other reasons	4	1.8 (0.5–4.0)	1	1.7 (0.0–6.4)
Fear of impaired fertility	1	0.5 (0.0–1.5)	0	0.0 (0)
No reason mentioned	45	20.6 (17.3–29.0)	12	20.3 (12.5–36.7)

In 81.3% of patients (*n* = 170) who decided against adjuvant treatment, reasons for refusing adjuvant therapy were documented. Multiple answers were possible.

**Table 3:** Adjusted relative risks with 95% confidence intervals for the associations between various exposure variables and decision for adjuvant treatment: Results from log-binomial regression models with complete case analysis and use of multiple imputation.

Exposure Variable	Number of Patients (%)		Adjuvant Treatment		RR_adj_ (95% CI) CC ^a^	RR_adj_ (95% CI) MI ^a^
	*n*	%	*n*	%	-	-
-	904		695	76.9	-	-
Age						
≤65 years (ref.)	460	50.9	406	88.3	1	-
>65 years	444	49.1	289	65.1	0.74 (0.68–0.80)	-
Gender						
Female (ref.)	378	41.8	290	76.7	1	-
Male	526	58.2	405	77.0	1.00 (0.93–1.08)	-
Charlson comorbidity index						
0 (ref)	621	68.7	501	80.7	1	1
≥1	283	31.3	194	68.6	0.99 (0.92–1.07)	0.99 (0.93–1.05)
Tumor stage						
IIIA (ref.)	81	9.0	58	71.6	1	1
IIIB	292	32.3	226	77.4	1.06 (0.96–1.16)	1.04 (0.96–1.13)
IIIC	426	47.1	329	77.2	1.05 (0.96–1.15)	1.04 (0.96–1.13)
IIID	26	2.9	23	88.5	1.07 (0.86–1.34)	1.07 (0.89–1.30)
IV ^b^	79	8.7	59	74.7	1.03 (0.90–1.17)	1.04 (0.93–1.16)
*BRAF* status						
Wild type (ref.)	471	52.1	354	75.2	1	-
Mutated	320	35.4	267	83.4	1.02 (0.97–1.07)	-
Prior intratumoral/adjuvant systemic treatment						
No (ref.)	782	86.5	588	75.2	1	1
Yes	122	13.5	107	87.7	1.04 (0.95–1.14)	1.03 (0.97–1.09)
Autoimmune disease						
No (ref.)	847	93.7	650	76.7	1	-
Yes	57	6.3	45	79.0	1.00 (0.93–1.08)	-
Region						
Northern Germany (ref.)	444	49.1	337	75.9	1	-
Southern Germany	460	50.9	358	77.8	1.03 (0.95–1.10)	-
Insurance status ^c^						
Statutory health insurance (ref.)	594	65.7	446	75.1	1	-
Private health insurance	127	14.0	103	81.1	1.08 (0.98–1.19)	1.08 (0.98–1.19)

Abbreviations: CC, complete case analysis; MI, multiple imputation; ref., reference; RR_adj_, adjusted relative risks with 95% confidence intervals for the associations between various exposure variables and decision for adjuvant treatment. ^a^ Different adjustment sets were applied for different exposure variables depending on the respective DAGs. Multiple imputation was performed for DAGs with covariates or confounder with incomplete data (insurance status; Figure S2). ^b^ Approval for adjuvant treatment in tumor stage IV was exclusively for ICI (nivolumab). ^c^ Insurance status data missing (*n* = 183).

**Table 4:** Patient characteristics of the cohort of *BRAF*-mutant patients with adjuvant treatment.

Characteristics	*BRAF*-Mutant Patients ^a^		PD-1 Blocker		Targeted Therapy		*p*-Value ^b^
	*n* 263	% 100	*n* 139	% (95% CI) 52.9 (46.8–59.2)	*n* 124	% (95% CI) 47.1 (40.8–53.2)	0.42
Median age (range)	56.3 (21.0–87.4)	-	57.4 (25.8–84.7)	(55–60)	55.6 (21.0–87.4)	(53–58)	-
Gender	-	-	-	-	-	-	0.45
Female	104	39.5	52	50.0 (40.4–60.0)	52	50.0 (40.4–60.0)	-
Male	159	60.5	87	54.7 (47.0–62.6)	72	45.3 (37.4–53.0)	-
Charlson comorbidity index	-	-	-	-	-	-	0.73
0	202	76.8	108	53.5 (46.7–60.1)	94	46.5 (39.9–53.3)	-
1–2	54	20.5	26	48.1 (34.5–62.2)	28	51.9 (37.8–65.5)	-
3–4	6	2.3	4	66.7 (*n*.e.)	2	33.3 (n.e.)	-
>5	1	0.4	1	100.0 (n.e.)	0	(n.e.)	-
Tumor stage	-	-	-	-	-	-	0.56
IIIA	24	9.1	14	58.3 (37.0–78.3)	10	41.7 (21.7–63.0)	-
IIIB	92	35.0	53	57.6 (47.9–68.4)	39	42.4 (31.6–52.1)	-
IIIC	136	51.7	67	49.3 (41.2–57.0)	69	50.7 (43.0–58.8)	-
IIID	11	4.2	5	45.5 (14.3–75.0)	6	54.5 (25.0–85.7)	-
IV	0	0.0	0	0.0 (n.e.)	0	0.0 (n.e.)	-
Prior intratumoral/adjuvant systemic treatment	-	-	-	-	-	-	0.02
No	224	85.2	125	55.8 (49.1–62.2)	99	44.2 (37.8–50.9)	-
Yes	39	14.8	14	35.9 (21.4–52.6)	25	64.1 (47.4–78.6)	-
Interferon	30	11.4	12	40.0	18	60	-
Dabrafenib/trametinib	3	1.1	2	66.6	1	33.3	-
Ipilimumab	1	0.4	0	0.0	1	100	-
Anti-PD-1	2	0.8	0	0.0	2	100	-
Intratumoral injection ^c^	3	1.1	0	0.0	3	100	-
Lymphadenectomy	-	-	-	-	-	-	0.22
Yes	125	47.5	71	56.8 (48.0–65.6)	54	43.2 (34.4–52.0)	-
No	138	52.5	68	49.3 (41.2–58.3)	70	50.7 (41.7–58.8)	-
Adjuvant radiotherapy	-	-	-	-	-	-	0.11
Yes	42	16.0	27	64.3 (50.0–78.6)	15	35.7 (21.4–50.0)	-
No	221	84.0	112	50.7 (43.9–57.3)	109	49.3 (42.7–56.1)	-
Autoimmune disease	-	-	-	-	-	-	0.02
Yes	17	6.5	4	23.5 (5.3–46.7)	13	76.5 (53.3–94.7)	-
No	246	93.5	135	54.9 (48.5–61.2)	111	45.1 (38.8–51.5)	-
Region ^d^	-	-	-	-	-	-	<0.01
Northern Germany	142	54.0	59	41.5 (34.2–51.4)	83	58.5 (48.6–65.8)	-
Southern Germany	121	46.0	80	66.1 (57.0–74.5)	41	33.9 (25.5–43.0)	-
Insurance status	-	-	-	-	-	-	0.43
Statutory health insurance	148	56.3	73	49.3 (41.0–57.7)	75	50.7 (42.3–59.0)	-
Private health insurance	42	16.0	24	57.1 (41.0–72.0)	18	42.9 (27.7–59.0)	-
Insurance status missing	73	27.8	42	57.5 (45.4–69.0)	31	42.5 (31.0–55.0)	-

Abbreviations: n.e., not estimable. ^a^ Data was enriched by 13 patients from one center that could not supply data of patients who decided against adjuvant therapy. ^b^ *t*-test for continuous variables, two-sided χ2 for categorical variables or Fisher´s exact tests (*n* < 5), as appropriate. ^c^ Intratumoral injection with interleukin-2, talimogene laherparepvec. ^d^ Northern German skin cancer centers: Kiel, Hamburg, Buxtehude, Hannover, Dortmund, Essen, Düsseldorf, Dresden; Southern German skin cancer centers: Mainz, Heidelberg, Erlangen, Würzburg, Tübingen.

**Table 5:** Adjusted relative risks with 95% confidence intervals for the associations between various exposure variables and decision for targeted therapy in *BRAF*-mutant patients: Results from log-binomial regression models with complete case analysis and use of multiple imputation.

Exposure Variable	Number of Patients (%)		Targeted Therapy		RR_adj_ (95% CI) CC ^a^	RR_adj_ (95% CI) MI ^a^
	*n*	%	*n*	%	-	-
-	263	-	124	47.1	-	-
Age						
≤65 years (ref.)	187	71.7	89	47.6	1	-
>65 years	76	28.9	35	46.1	0.97 (0.73–1.29)	-
Gender						
Female (ref.)	104	39.5	52	50.0	1	-
Male	159	60.5	72	45.3	0.91 (0.70–1.17)	-
Charlson comorbidity index						
0 (ref)	202	76.8	94	46.5	1	1
≥1	61	23.2	30	49.2	1.17 (0.79–1.75)	1.12 (0.80–1.56)
Tumor stage						
IIIA (ref)	24	9.1	10	41.7	1	1
IIIB	92	35.0	39	42.4	0.76 (0.45–1.26)	1.05 (0.61–1.80)
IIIC	136	51.7	69	50.7	1.00 (0.61–1.64)	1.29 (0.76–2.18)
IIID	11	4.2	6	54.6	1.09 (0.44–2.72)	1.46 (0.71–3.03)
Prior intratumoral/adjuvant systemic treatment						
No (ref.)	224	85.2	99	44.2	1	1
Yes	39	14.8	25	64.1	1.24 (0.93–1.68)	1.24 (0.93–1.68)
Autoimmune disease ^c^						
No (ref.)	246	93.5	111	45.1	1	-
Yes	17	6.5	13	76.5	1.73 (1.25–2.39)	-
Region						
Northern Germany (ref.)	142	54.0	83	58.5	1	-
Southern Germany	121	46.0	41	33.9	0.58 (0.44–0.77)	-
Insurance status ^b^						
Statutory health insurance (ref.) ^c^	148	56.3	75	50.7	1	1
Private health insurance	42	16.0	18	42.9	0.85 (0.58–1.24)	0.81 (0.55–1.20)

Therapy choice in *BRAF*-mutated patients who opted for adjuvant treatment. Data was enriched by 13 patients from one center that could not supply data of patients who decided against adjuvant therapy. Abbreviations: CC, complete case analysis; MI, multiple imputation; RR_adj_, adjusted relative risks with 95% confidence intervals for the associations between various exposure variables and decision for targeted therapy in *BRAF*-mutant patients. ^a^ Different adjustment sets were applied for different exposure variables depending on the respective DAGs. Multiple imputation was performed for DAGs with covariates or confounder with incomplete data (insurance status; Figure S3). ^b^ Approval for adjuvant treatment in tumor stage IV was exclusively for ICI (nivolumab). ^c^ Insurance status data missing (*n* = 77).

## Data Availability

Data sets supporting reported results are archived. In case of interest, contact the corresponding author.

## Supplementary Materials

The following are available online at https://www.mdpi.com/article/10.3390/cancers13102319/s1, Figure S1. Participating skin cancer centers of the German Dermatologic Cooperative Oncology Group, Figure S2. A–H: Directed acyclic graph (DAG) determining confounding covariates for decision for adjuvant treatment in total cohort, Figure S3. A–I: Directed acyclic graph (DAG) determining confounding covariates for decision for targeted therapy in *BRAF*-mutated patients, Figure S4. Flowchart for the selection of the study population. 13 skin cancer centers: Essen, Tübingen, Hannover, Dresden, Buxtehude, Kiel, Würzburg, Mainz, Erlangen, Hamburg, Dortmund, Düsseldorf, Heidelberg, Figure S5. Percentage distribution for adjuvant treatment decision per single skin cancer center, Figure S6. Percentage distribution per skin cancer center for decision for (a) ICI in *BRAF* wild-type patients/*BRAF* mutation status unknown; ^a^ *BRAF* mutation status not specified before start of adjuvant treatment; ^b^ (in total *n* = 450), (b) ICI in *BRAF*-mutant patients (*n* = 156), and (c) TT in *BRAF*-mutant patients (*n* = 124). Distribution per single skin center, Table S1. Comorbidities of the modified Charlson comorbidity index by Quan et al., Med Care 2005 [19], Table S2. Reasons against adjuvant treatment as given by patients for the total cohort and divided by gender and age.
