# Supplementary material for: Factors Influencing the Adjuvant Therapy Decision: Results of a Real-World Multicenter Data Analysis of 904 Melanoma Patients
Source: Cancers (Basel). 2021 May 12;13(10):2319. doi: 10.3390/cancers13102319 (PMC8151445; doi:10.3390/cancers13102319)
Supplement: Supplementary file 1 [file cancers-13-02319-s001.zip › cancers-1192266-SI.pdf]

cancers

MDPI

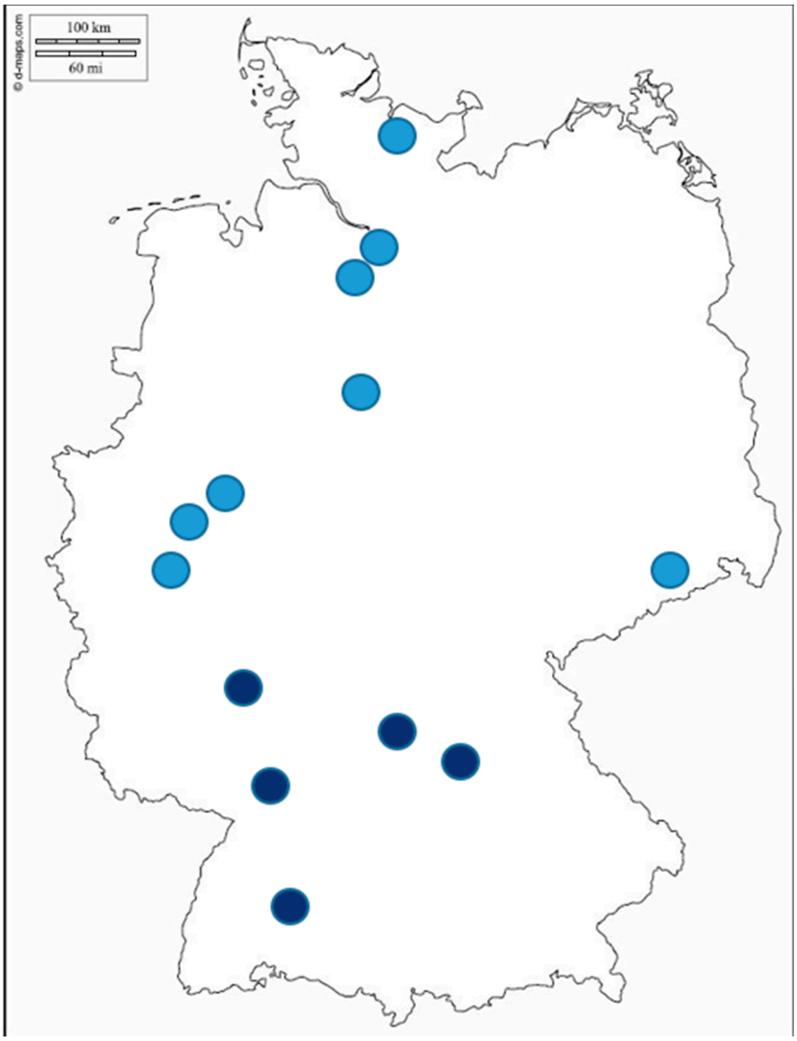

Figure S1. Participating skin cancer centers of the German Dermatologic Cooperative Oncology Group. Northern German skin cancer centers (n = 444 patients): Kiel, Hamburg, Buxtehude, Hannover, Dortmund, Essen, Düsseldorf, Dresden. Southern German skin cancer centers (n = 460 patients): Mainz, Heidelberg, Erlangen, Würzburg, Tübingen. Adapted to: https://d-maps.com/m/europa/germany/allemagne_de/allemagne_de42.pdf, modified. Permission to use the map has been granted, 23 December 2020.

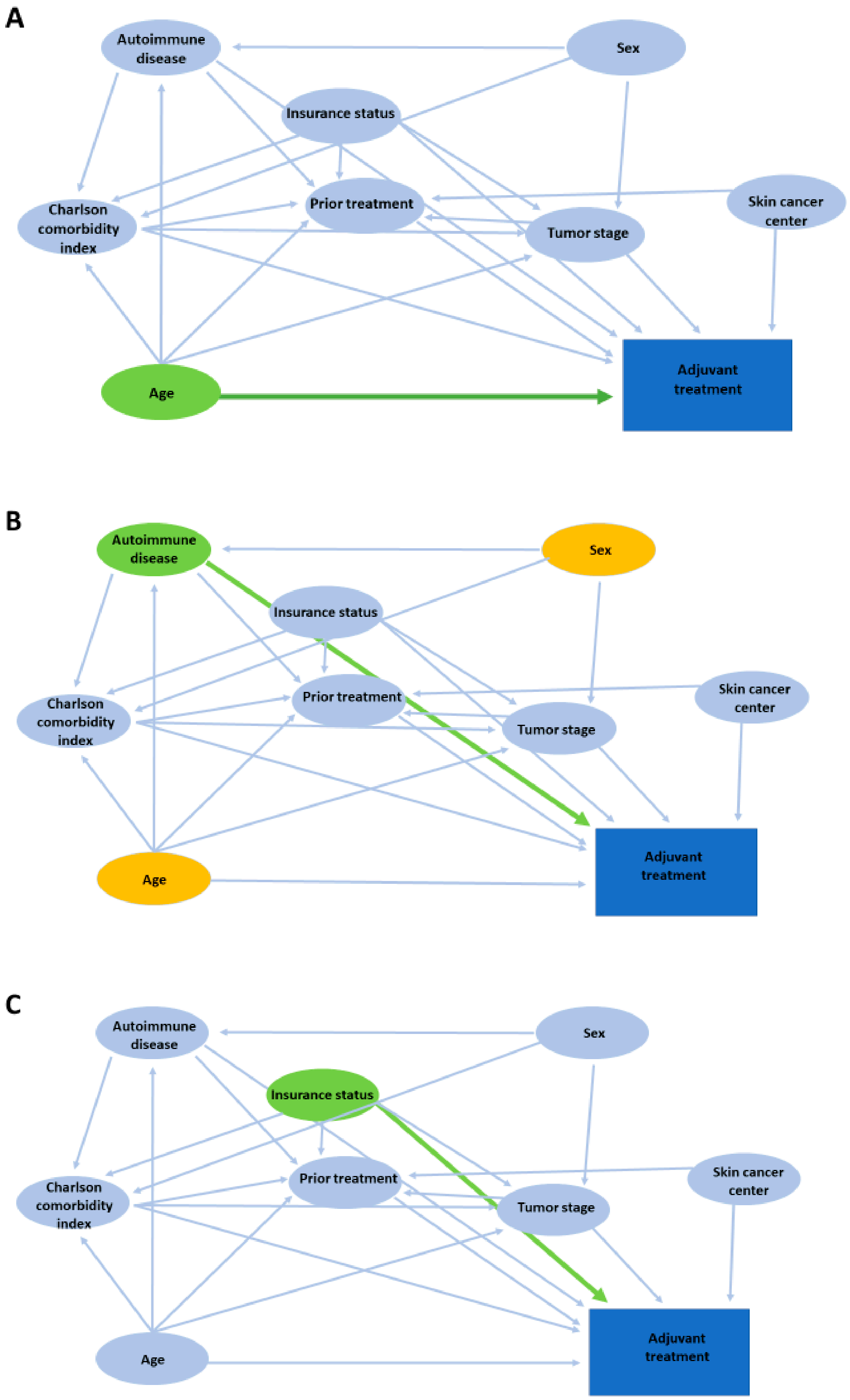
A
Autoimmune disease
Sex
Insurance status
Charlson comorbidity index
Prior treatment
Skin cancer center
Tumor stage
Age
Adjuvant treatment
B
Autoimmune disease
Sex
Insurance status
Charlson comorbidity index
Prior treatment
Skin cancer center
Tumor stage
Age
Adjuvant treatment
C
Autoimmune disease
Sex
Insurance status
Charlson comorbidity index
Prior treatment
Skin cancer center
Tumor stage
Age
Adjuvant treatment

D

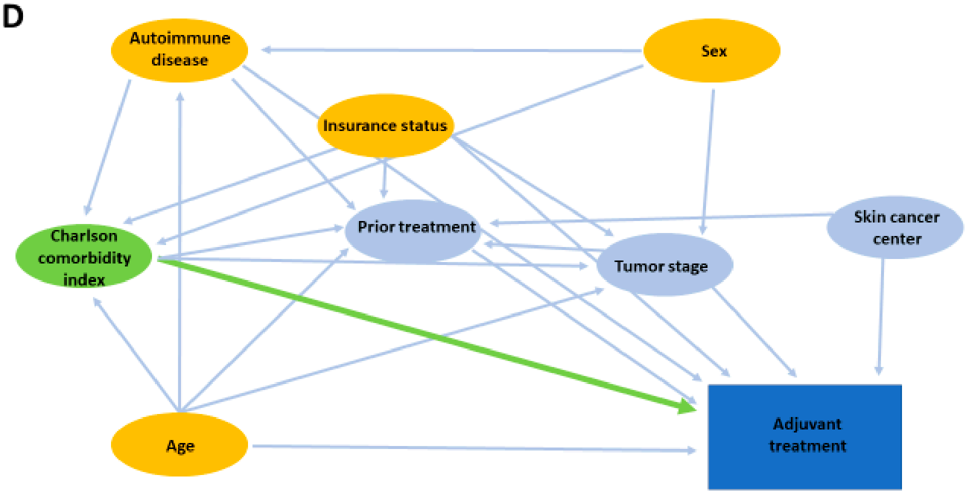
Autoimmune disease
Sex
Insurance status
Charlson comorbidity index
Prior treatment
Skin cancer center
Tumor stage
Age
Adjuvant treatment

E

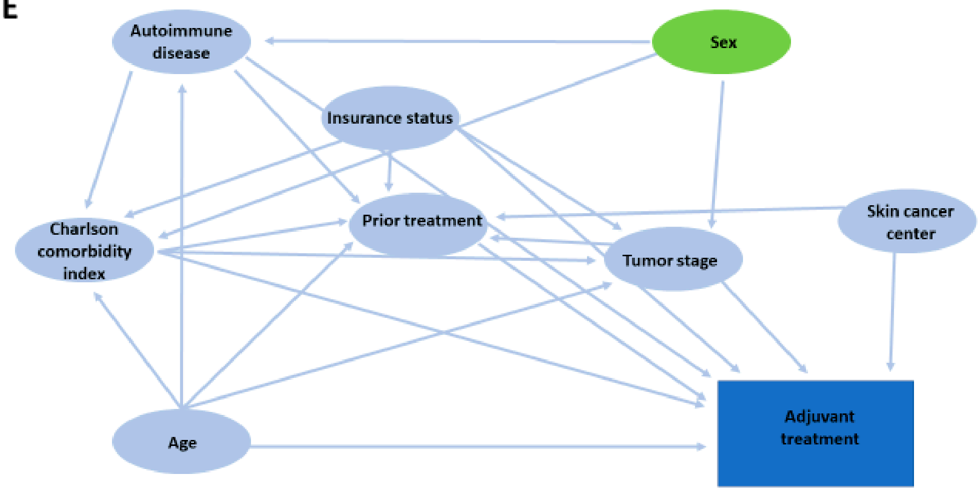
Autoimmune disease
Sex
Insurance status
Charlson comorbidity index
Prior treatment
Skin cancer center
Tumor stage
Age
Adjuvant treatment

F

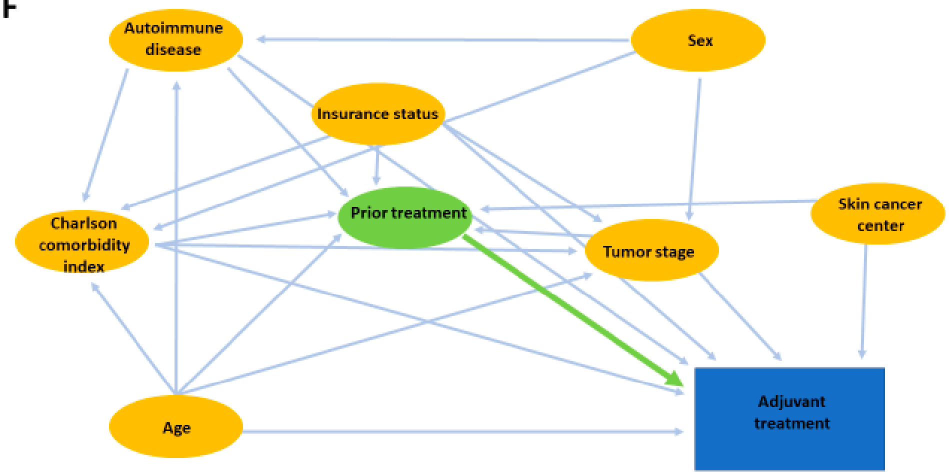
Autoimmune disease
Sex
Insurance status
Charlson comorbidity index
Prior treatment
Skin cancer center
Tumor stage
Age
Adjuvant treatment

G

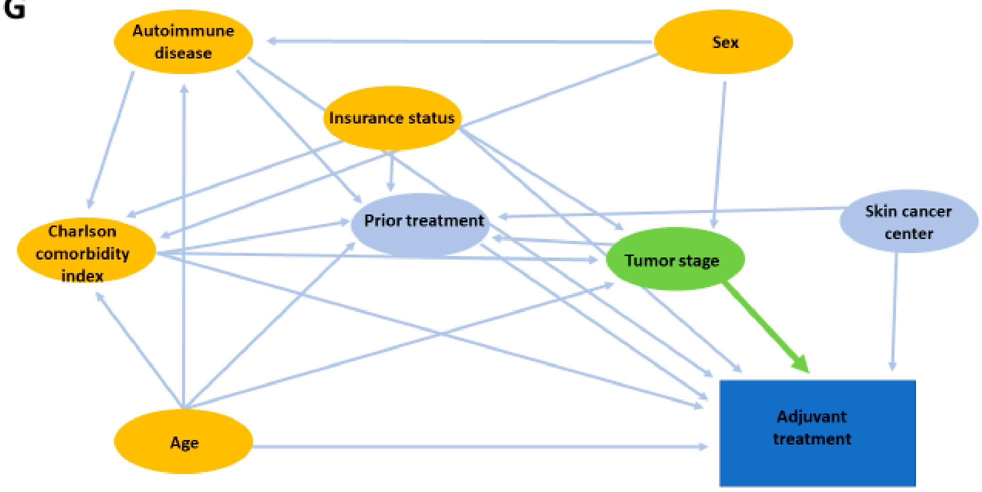

H

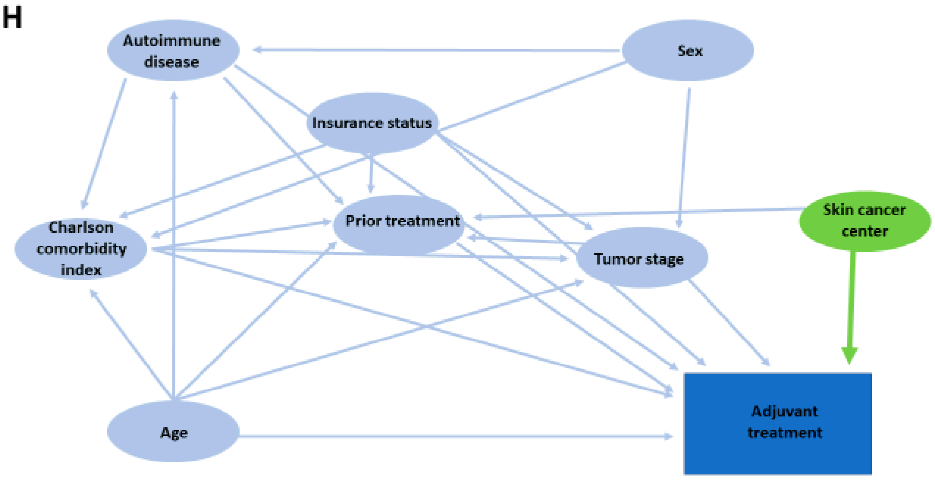

Figure S2. A–H: Directed acyclic graph (DAG) determining confounding covariates for decision for adjuvant treatment in total cohort. Exposure of interest: (**A**) age, (**B**) autoimmune disease, (**C**) insurance status, (**D**) Charlson comorbidity index, (**E**) gender, (**F**) prior treatment, (**G**) tumor stage, (**H**) skin cancer center.

A

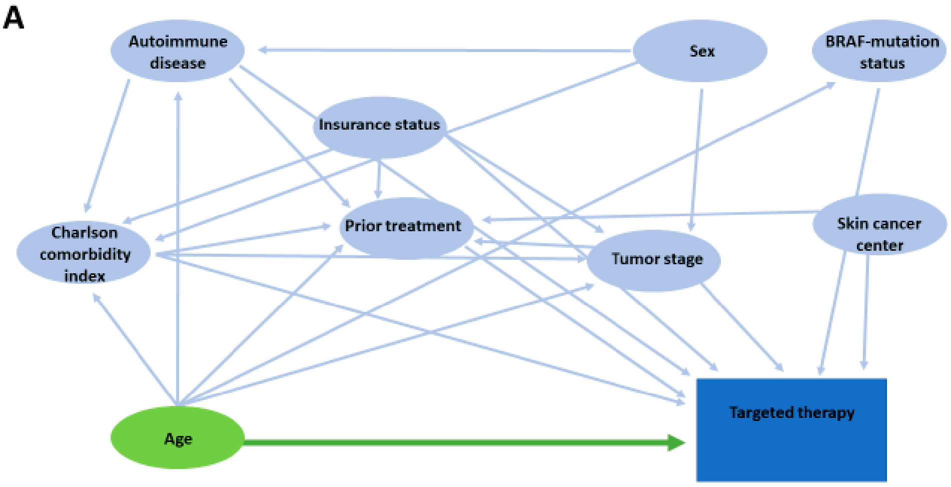

B

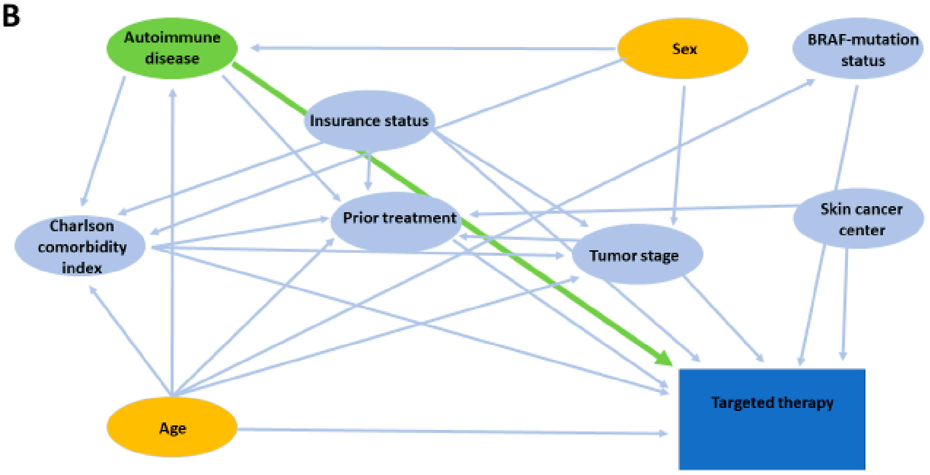
Autoimmune disease
Sex
BRAF-mutation status
Insurance status
Prior treatment
Skin cancer center
Charlson comorbidity index
Tumor stage
Targeted therapy
Age

C

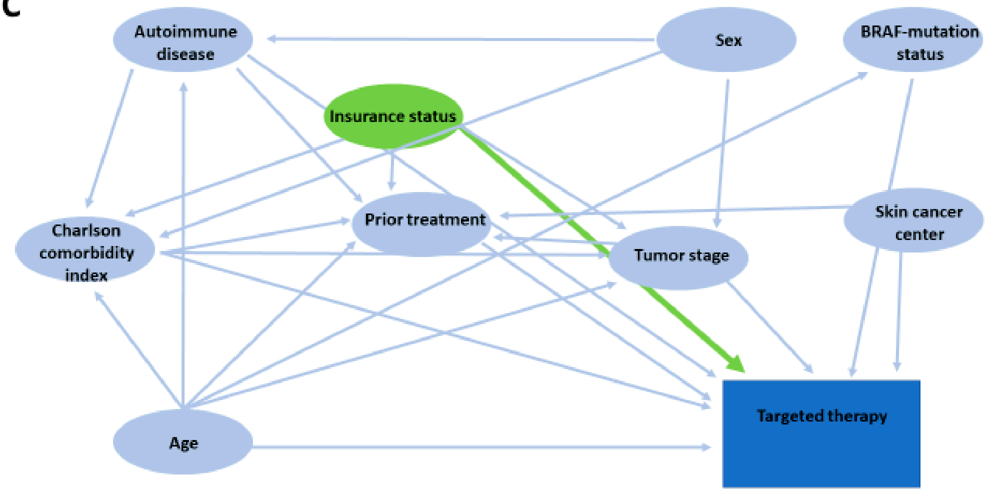
Autoimmune disease
Sex
BRAF-mutation status
Insurance status
Prior treatment
Skin cancer center
Charlson comorbidity index
Tumor stage
Targeted therapy
Age

D

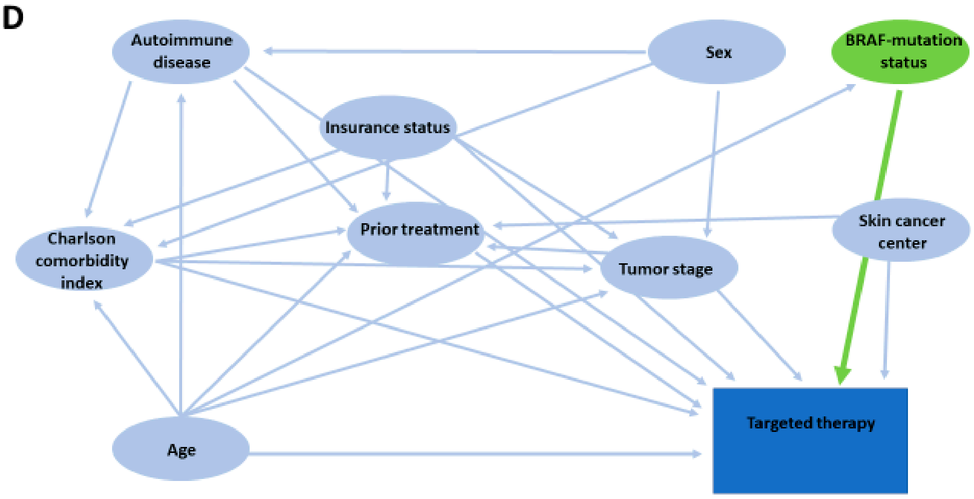
Autoimmune disease
Sex
BRAF-mutation status
Insurance status
Prior treatment
Skin cancer center
Charlson comorbidity index
Tumor stage
Targeted therapy
Age

E

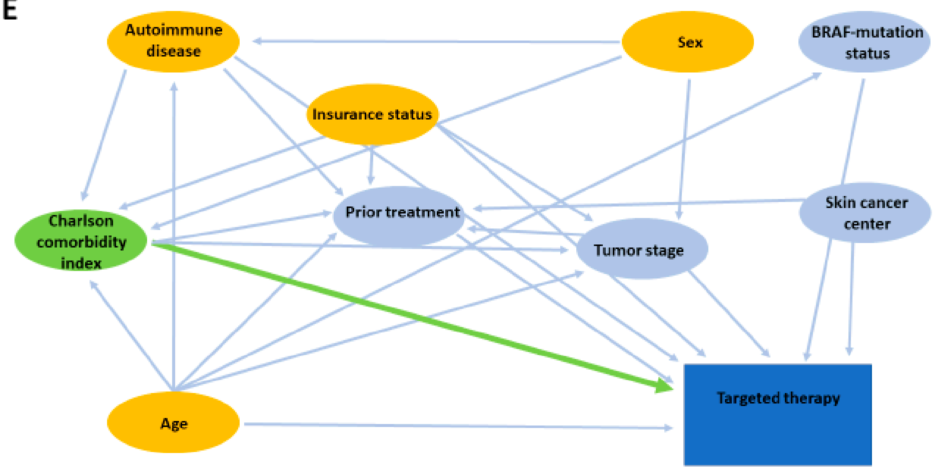

Autoimmune disease
Sex
BRAF-mutation status
Insurance status
Charlson comorbidity index
Prior treatment
Skin cancer center
Tumor stage
Age
Targeted therapy

F

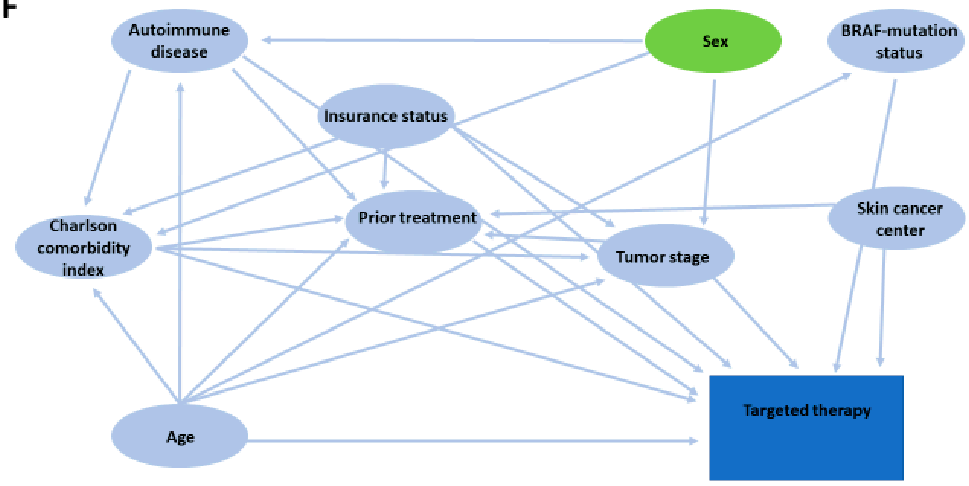

Autoimmune disease
Sex
BRAF-mutation status
Insurance status
Charlson comorbidity index
Prior treatment
Skin cancer center
Tumor stage
Age
Targeted therapy

G

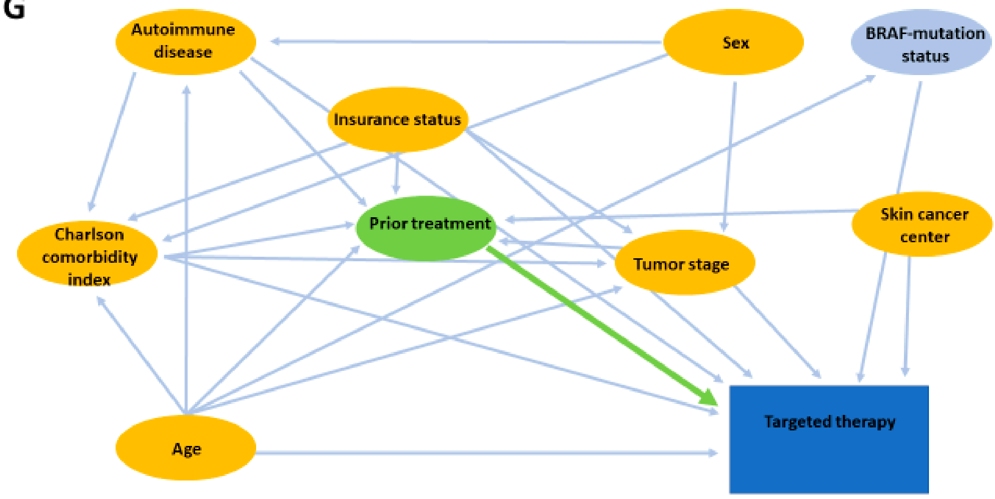

Autoimmune disease
Sex
BRAF-mutation status
Insurance status
Charlson comorbidity index
Prior treatment
Skin cancer center
Tumor stage
Age
Targeted therapy

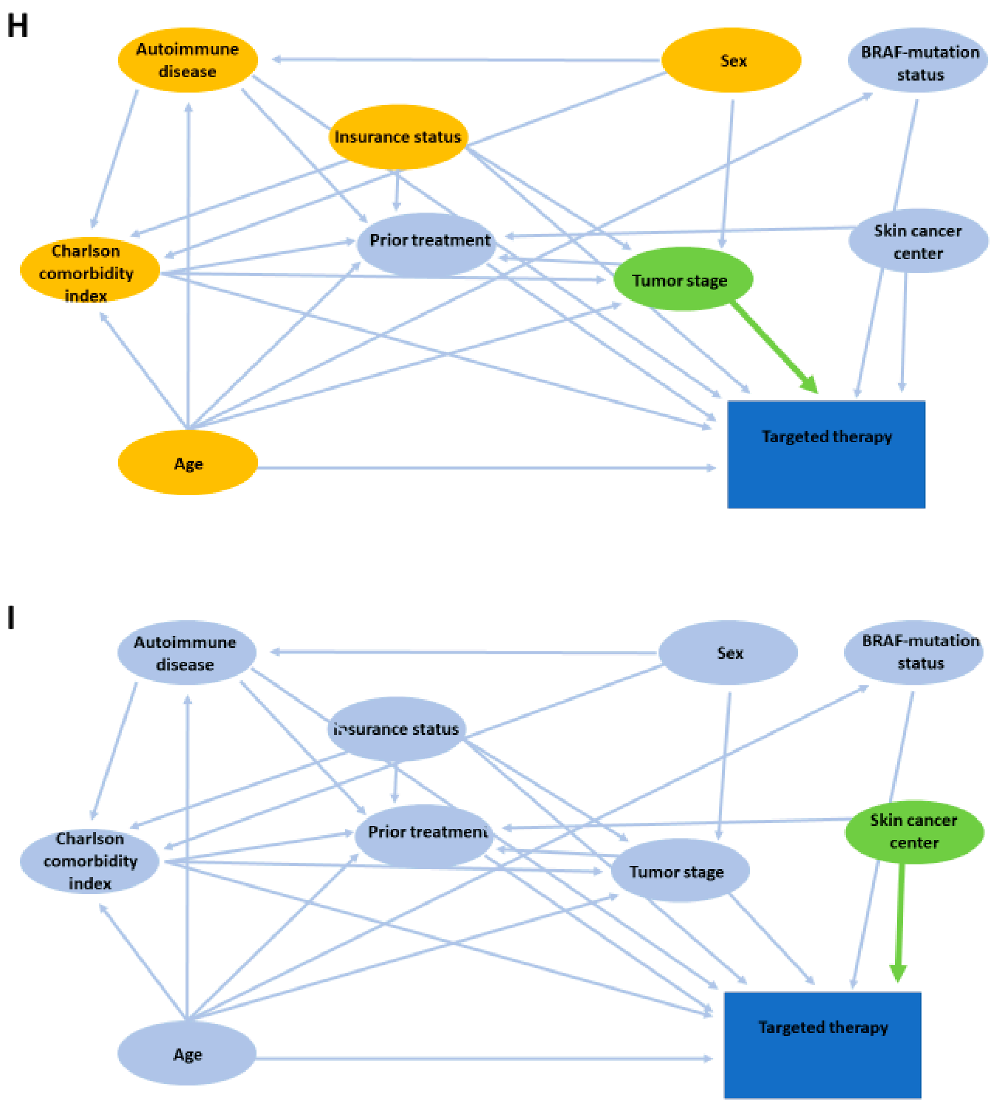

Figure S3. A–I: Directed acyclic graph (DAG) determining confounding covariates for decision for targeted therapy in *BRAF*-mutated patients. (**A**) age, (**B**) autoimmune disease, (**C**) insurance status, (**D**) *BRAF*-mutation status, (**E**) Charlson comorbidity index, (**F**) gender, (**G**) prior treatment, (**H**) tumor stage, (**I**) skin cancer center.

Figures S2 and S3: Directed acyclic graphs (DAGs) used to determine the confounding covariates for the decision on adjuvant treatment (yes vs. no) in total cohort, and for of targeted therapy (yes vs. no) in *BRAF*-mutated patients. Correlations were developed by consensus of melanoma experts from several skin cancer centers and empirical literature research. The exposures of interest are marked in green (age, autoimmune disease, insurance status, *BRAF*-mutation status, Charlson comorbidity index, gender, prior treatment, tumor stage, skin cancer centers); covariates are marked in light blue; confounders are marked in orange. Interactions between covariates are indicated by arrows. DAGs were built for decision for or against adjuvant treatment in total cohort (Figure S2) and decision for TT in *BRAF*-mutated patients (Figure S3). The resulting confounders depended on the respective exposure of interest and include age (≤65 years vs >65 years), autoimmune disease (no vs. yes), insurance status (statutory health insurance vs. private health insurance), sex (female vs. male), Charlson comorbidity index (0 vs. ≥1), skin cancer center and tumor stage at time of decision for or against treatment. Due to missing data

of 220 of 904 patients for insurance status, additional Cox regression analyses accounting for multiple imputation of insurance status were performed.

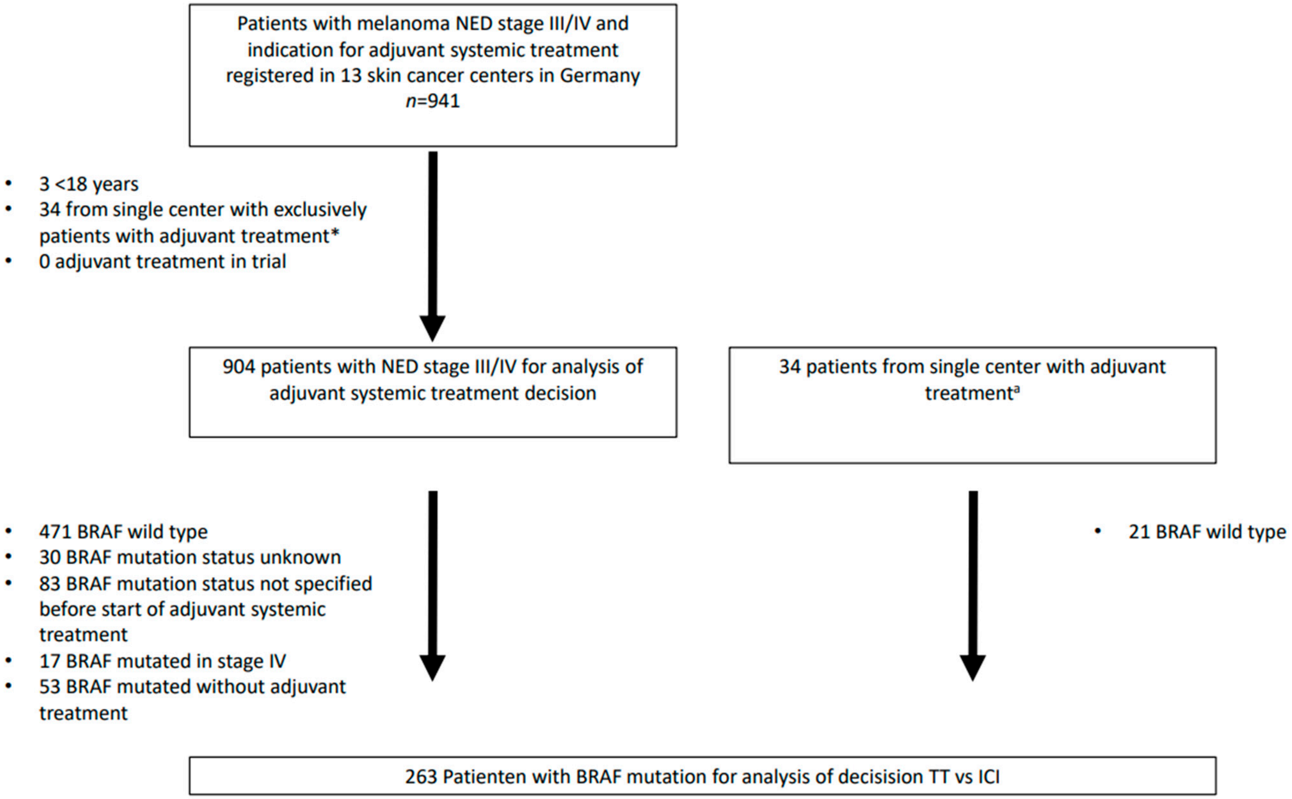

Figure S4. Flowchart for the selection of the study population from 13 skin cancer centers: Essen, Tübingen, Hannover, Dresden, Buxtehude, Kiel, Würzburg, Mainz, Erlangen, Hamburg, Dortmund, Düsseldorf and Heidelberg. [a] These patients were considered when evaluating the type of adjuvant therapy in *BRAF*-mutant patients. Abbreviations: NED, no evidence of disease.

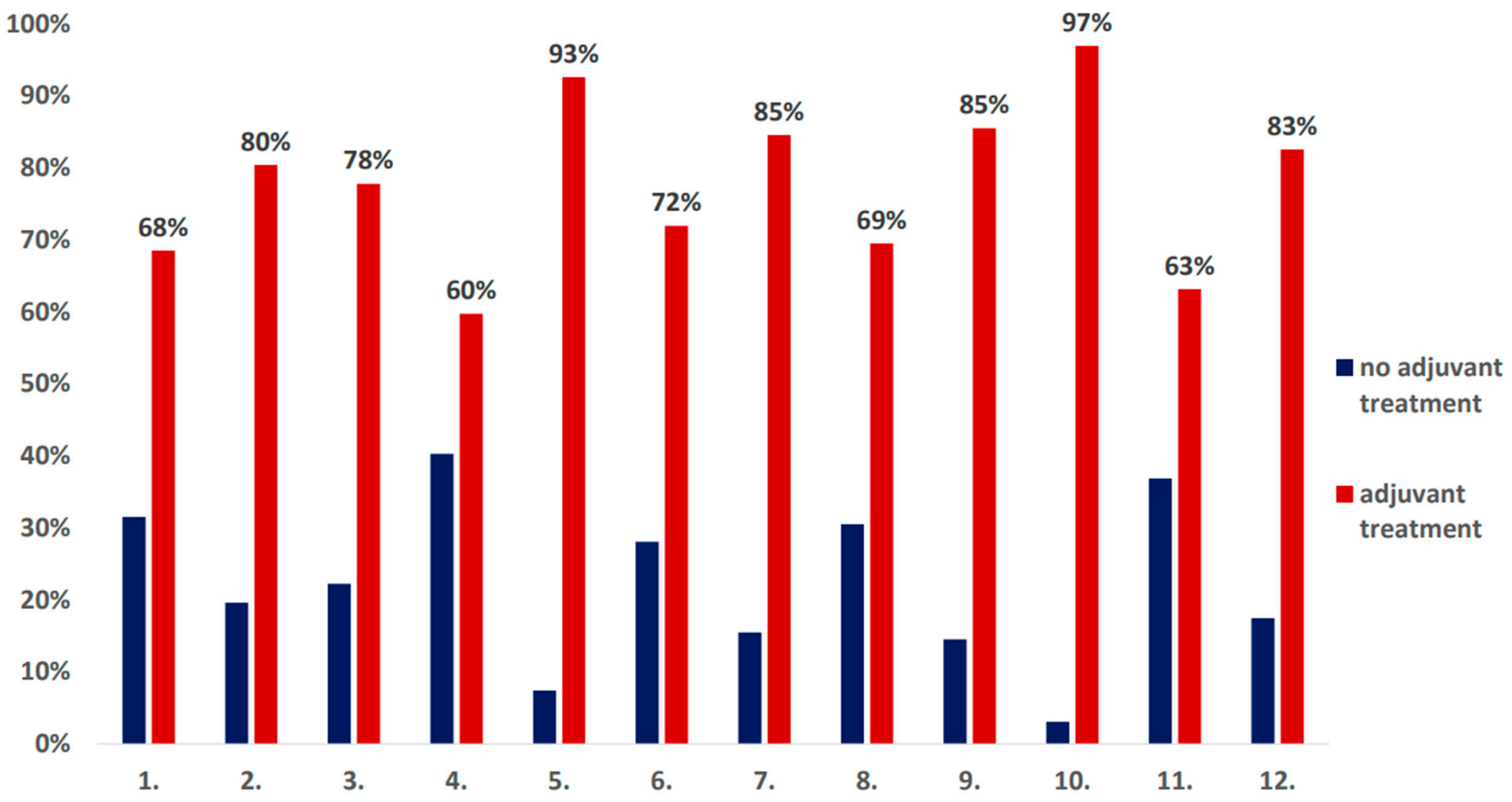

Figure S5. Percentage distribution for adjuvant treatment decision per single skin cancer center. Distribution per single skin cancer center. One center was excluded, as only data of patients who opted for adjuvant systemic treatment but not against systemic adjuvant treatment were submitted.

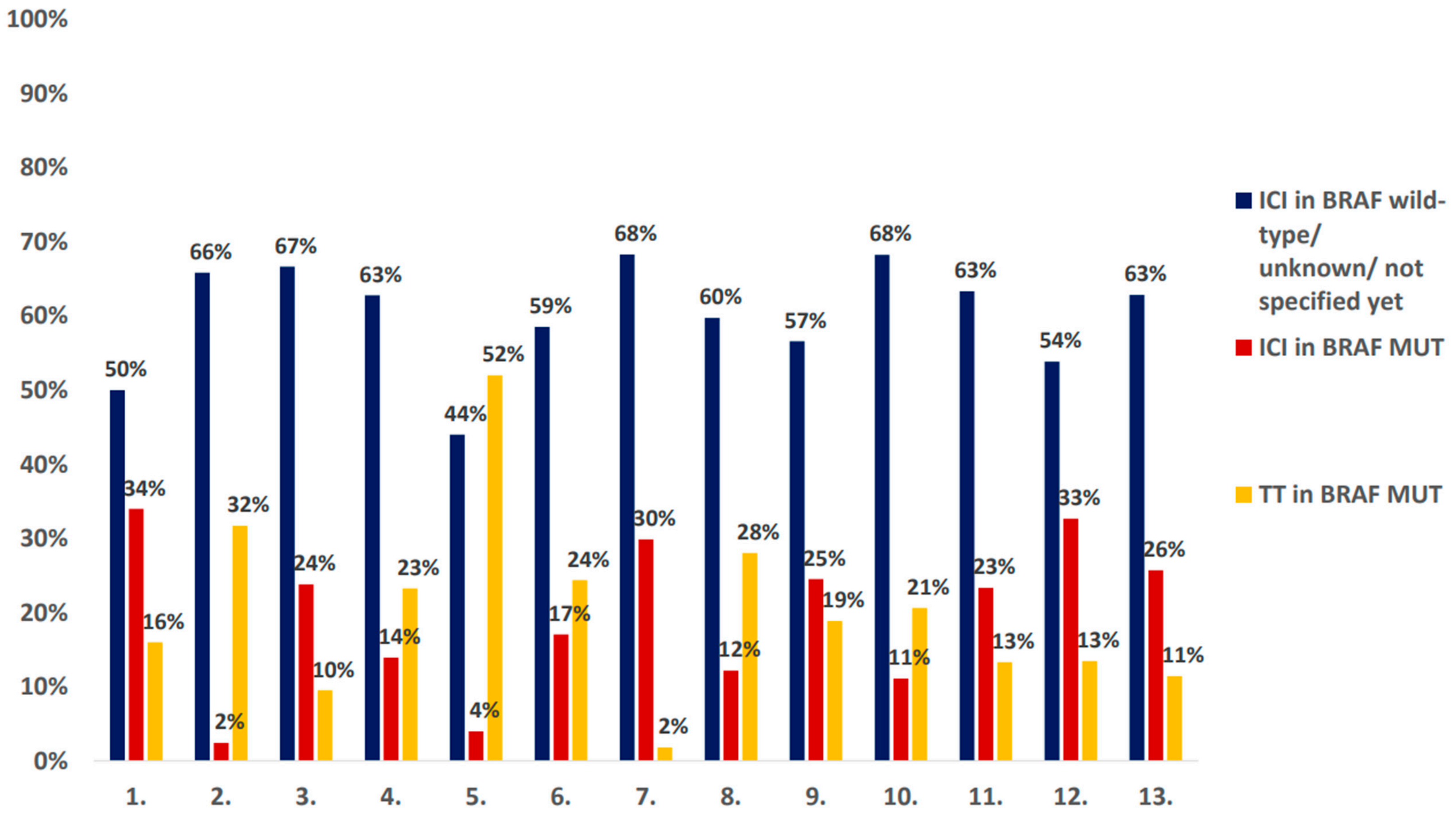

Figure S6. Percentage distribution per skin cancer center for decision for ICI in *BRAF* wild-type patients/*BRAF* mutation status unknown [a]/*BRAF* mutation status not specified before start adjuvant treatment [b] (in total *n* = 450); ICI in *BRAF*-mutant patients (*n* = 156); and TT in *BRAF* mutant patients (*n* = 124). Distribution per single skin center. [a] Decision for adjuvant therapy was made without prior molecular pathology result, and *BRAF* status was not routinely determined. [b] *BRAF* mutational status was only known after start of adjuvant therapy.

Table S1. Comorbidities of the modified Charlson comorbidity index by Quan et al., Med Care 2005 [19].

**Myocardial Infarction**	**I21.x, I22.x, I25.2**
Congestive heart failure	I09.9, I11.0, I13.0, I13.2, I25.5, I42.0, I42.5–I42.9, I43.x, I50.x, P29.0
Peripheral vascular disease	I70.x, I71.x, I73.1, I73.8, I73.9, I77.1, I79.0, I79.2, K55.1, K55.8, K55.9, Z95.8, Z95.9
Cerebrovascular disease	G45.x, G46.x, H34.0, I60.x–I69.x
Dementia	F00.x–F03.x, F05.1, G30.x, G31.1
Chronic pulmonary Disease	I27.8, I27.9, J40.x–J47.x, J60.x–J67.x, J68.4, J70.1, J70.3
Rheumatic disease	M05.x, M06.x, M31.5, M32.x–M34.x, M35.1, M35.3, M36.0
Peptic ulcer disease	K25.x–K28.x
Mild liver disease	B18.x, K70.0–K70.3, K70.9, K71.3–K71.5, K71.7, K73.x, K74.x, K76.0, K76.2–K76.4, K76.8, K76.9, Z94.4
Diabetes without chronic Complication	E10.0, E10.1, E10.6, E10.8, E10.9, E11.0, E11.1, E11.6, E11.8, E11.9, E12.0, E12.1, E12.6, E12.8, E12.9, E13.0, E13.1, E13.6, E13.8, E13.9, E14.0, E14.1, E14.6, E14.8, E14.9
Diabetes with chronic Complication	E10.2–E10.5, E10.7, E11.2–E11.5, E11.7, E12.2–E12.5, E12.7, E13.2–E13.5, E13.7, E14.2–E14.5, E14.7
Hemiplegia or paraplegia	G04.1, G11.4, G80.1, G80.2, G81.x, G82.x, G83.0–G83.4, G83.9
Renal disease	I12.0, I13.1, N03.2–N03.7, N05.2–N05.7, N18.x, N19.x, N25.0, Z49.0–Z49.2, Z94.0, Z99.2
Any malignancy, including lymphoma and leukemia, except malignant neoplasm of skin	C00.x–C26.x, C30.x–C34.x, C37.x–C41.x, C43.x, C45.x–C58.x, C60.x–C76.x, C81.x–C85.x, C88.x, C90.x–C97.x
Moderate or severe liver Disease	I85.0, I85.9, I86.4, I98.2, K70.4, K71.1, K72.1, K72.9, K76.5, K76.6, K76.7
Metastatic solid tumor	C77.x–C80.x
AIDS/HIV	B20.x–B22.x, B24.x

Table S2. Reasons against adjuvant treatment as given by patients for the total cohort and divided by sex and age. [a] Patient opinions, n = 170 patients, multiple answers possible. [b] no health insurance.

**Patients´ Reasons Against Adjuvant Treatment**	***n***	**%**	**Gender**				**Age**			
			**Female**		**Male**		**≤ 65 Years**		**>65 Years**	
			n	**%**	***n***	**%**	***n***	**%**	***n***	**%**
	**218** [a]	**100**	**95**	**43.6**	**123**	**56.4**	**56**	**25.7**	**162**	**74.3**
Age	64	29.4	25	26.3	39	31.7	4	7.1	60	37.0
Fear of adverse events	46	21.1	28	29.5	18	14.6	17	30.4	29	17.9
Quality of life	26	11.9	8	8.4	18	14.6	9	16.1	17	10.5

Too much effort	15	6.9	6	6.3	9	7.3	3	5.4	12	7.4
Low risk of recurrence (patient opinion)	11	5.0	4	4.2	7	5.7	7	12.5	4	2.5
Time interval initial diagnosis of melanoma and recurrence	6	2.8	2	2.1	4	3.3	4	7.1	2	1.2
Other reasons [b]	4	1.8	0	0.0	4	3.3	-	0.0	4	2.5
Fertility	1	0.5	1	1.1	0	0.0	1	1.8	0	0.0
No reason mentioned	45	20.6	21	22.1	24	19.5	11	19.6	34	21.0
